# A novel method for estimating subresolution porosity from CT images and its application to homogeneity evaluation of porous media

**DOI:** 10.1038/s41598-022-20086-x

**Published:** 2022-09-28

**Authors:** Li Zhuang, Hyu-Soung Shin, Sun Yeom, Chuyen Ngoc Pham, Young-Jae Kim

**Affiliations:** 1 Korea Institute of Civil Engineering and Building Technology, Goyang, Republic of Korea; 2 University of Science and Technology, Daejeon, Republic of Korea

**Keywords:** Civil engineering, Aerospace engineering, Computer science

## Abstract

We propose a new method, i.e., the statistical phase fraction (SPF) method, to estimate the total porosity and spatial distribution of local porosities from subresolution pore-dominated X-ray microtomography images of porous materials. The SPF method assumes that a voxel in a CT image is composed of either a single or a maximum of three pure phases of matter (solid, liquid and air). Gaussian function (GF) fitting is conducted on the basis that the summation of the area of each GF curve is equal to the total area covered by the CT histogram. The volume fraction of each phase corresponding to each GF is calculated based on the mean value of the GF, the area of the GF, and the CT numbers for pure phases. The SPF method is verified on three different types of components containing only air and solid phases, i.e., alumina ceramic and two sintered lunar regolith simulants with relatively homogenous and inhomogeneous microstructures. The estimated porosities of a total of 15 specimens (the total porosity ranges from 0 to 51%) via the SPF method show an average error of 3.11% compared with the ground truth. Spatial distribution of local porosities in the defined representative element volume is investigated for homogeneity evaluation. Results show that the local porosity inhomogeneity in the sintered FJS-1 specimens is more prominent than that in the sintered KLS-1 specimens.

## Introduction

Porous materials are ubiquitous, i.e., from ceramics to soils and rocks to human bone and various components. These materials are everywhere in daily life and are critical to virtually medical and all industrial construction and energy processes. Many construction materials are porous, such as typical concrete and cement. In recent years, with increasing interest in the lunar exploration and base construction, building materials manufactured through sintering of the in-situ resources (i.e., lunar regolith) and the characterization of sintered porous materials have attracted increasing attention^1–5^. Evaluation on the porosity, homogeneity or heterogeneity is very important, as the matrix (structure) determines the physical and mechanical behavior, such as thermal conductivity, permeability and strength. In addition, material characterization of the sintered specimens helps to determine the sintering process and equipment design.

Most porous materials are composed of matrix and pores at the microscale or nanoscale^6^. X-ray computed tomography (CT), in particular the industrial X-ray CT^7^, as a nondestructive technique, has been widely used to analyze porous materials. Major advantage of this technique include the quantitative measurement of pore structures, such as local and total porosities, pore size distribution and pore shapes, and analysis of microstructural evolution during processing^8–10^. To separate the pores and solid matrix in CT images, segmentation must be performed. A traditional and the simplest segmentation technique is the thresholding method, where a threshold pixel intensity value is selected to divide the image into two portions and pores are usually grouped into the portion that has pixel values smaller than the threshold^11,12^. There is a trade-off between sample size and resolution. In many cases, image resolution is limited depending on the required representative elementary volume (REV) for the porosity estimation and geometric properties of the sample, and this results in that the pore size of the samples being below the image resolution. This type of pore is therefore defined as a subresolution pore. For a subresolution porosity estimation, pore segmentation using the threshold-based approach has difficulties because the approach can only deal with pore sizes that are greater than or equal to 1 pixel or voxel. A combined CT scans and focused ion beam–scanning electron microscopy (FIB–SEM) imaging technique has been applied to capture pores in dolomite rock at the micrometer and nanometer scales^13^. However, this method requires millimeter-sized samples, which is not feasible for every material. In addition, data acquisition via FIB-SEM analysis on a very limited volume takes longer, and therefore, there is no advantage to utilizing this method on materials that are highly nonuniform or have a large volume. In previous studies, the differential imaging method was employed where the sample is scanned under both dry and fully liquid saturated conditions, and the differential image between the two is used for segmentation^14–16^. Additionally, specific liquids, e.g., high-salinity brine, potassium iodide solution and mercury, have been used to enhance image contrast^17^. However, this method does not apply to all cases, as liquid saturation could disturb the sample. In addition, many samples contain closed pores that are hard to saturate. In the past two decades, X-ray and neutron dark-field imaging (DFI) have been frequently explored as techniques to overcome the trade-off between spatial resolution and field-of-view (FOV) and, in particular, to detect subresolution features such as pores or fractures^18,19^. While the DFI method has been reported to be able to differentiate between two groups of large and small subresolution pores, validation of the method in a wide range of nonuniform subresolution pores has not been established, and quantitative evaluation of subresolution porosity using the method is questionable.

An intravoxel analysis method was reported for the evaluation of voxel-specific microporosity by employing the linear relationship between gray values and the X-ray attenuation coefficient^20^, which is dependent on the effective X-ray energy and can be obtained from a National Institute of Standards and Technology (NIST) database^21^. This method was validated on a typical ceramic biomaterial for engineering scaffolds^20^ and fired clay ceramic specimens^22^ to estimate the voxel-specific microporosity. Furthermore, the macroporosity of the specimens was measured by traditional threshold-based image analysis. A two-step segmentation algorithm for predicting the porosity of single-mineral rocks that are heterogeneous and contain a large fraction of subresolution pores has been reported^23^. Using this segmentation method, the pure solid, pure voids and residual phases were separated, and the porosity of the residual phase was calculated with respect to the two pure phases, i.e., porosity values of 0 (for the solid) and 1 (for voids)^23^. There are a few similarities between the method in the reference^23^ and our method in terms of the phase concept (e.g., 0 for solid and 1 for voids), while the algorithm and estimation process on the CT histogram and porosity analysis are completely different.

In this study, we present a new method, called the statistical phase fraction (SPF) method, which was developed and implemented from its original concept firstly proposed in 2013^24^, to estimate the total porosity of porous samples, particularly those containing subresolution pores, via CT histogram analysis. In particular, the SPF method is different from the existing methods in that it can estimate porosity for an arbitrary local part of a given sample without destroying the sample. Through the analysis of local porosities, the SPF method is applied to evaluate the homogeneity of sintered specimens from two kinds of lunar regolith simulant.

## Concept of the proposed method

**Figure 1:**
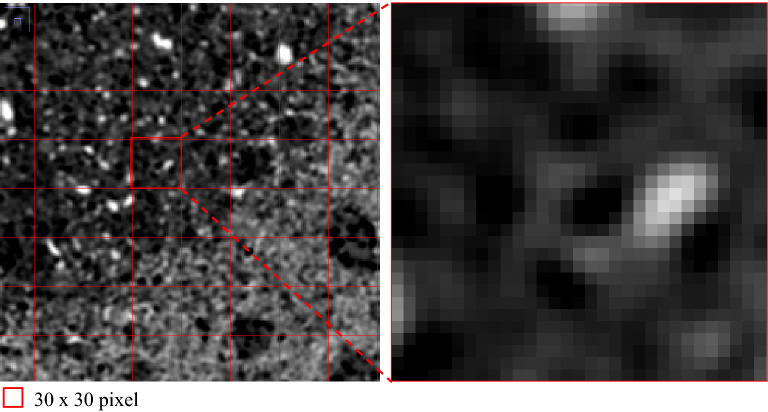
A typical cross-sectional CT image of a porous sample of sintered lunar regolith simulant consisting of pixels with different gray intensity values.

**Figure 2:**
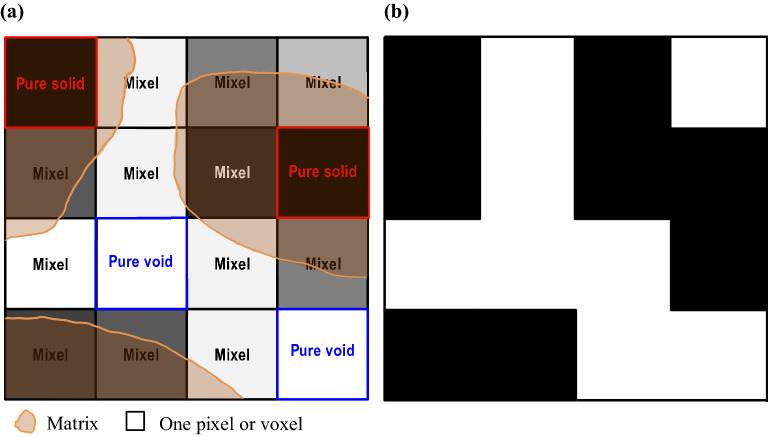
The “Mixel” concept in (**a**) the raw image compared with (**b**) the “black” and “white” voxels in traditional binarization via the thresholding method.

The fundamental principle of CT is that the attenuation of an X-ray beam passing through an object can be recorded and processed as signals, and later, these signals can be converted into digital images of pixel or voxel arrays, corresponding to 2D and 3D, respectively. This process is referred to as CT reconstruction^25^. Each voxel has an intensity value that represents the X-ray attenuation property (e.g., Fig. 1). These voxels represent different components in the sample, which are typically classified into the three common phases of solid, liquid and air. For porosity estimations, the traditional method requires segmentation of the void phase and the other phases, and voids can be either occupied by air or liquid. Successful segmentation requires the dimensions of the target material components to be larger than the image resolution, i.e., segmentation is possible only when the material components or phases can be distinguished visually on the CT image. This is clearly impossible for the estimation of subresolution porosity, where pores are smaller than a single pixel in 2D or a voxel in 3D, which cannot be recognized from the image. Therefore, we introduce a new term called “Mixel” which represents a pixel or a voxel consisting of two or more phases. As shown in Fig. 2, the concept of a “Mixel” was explained assuming matrix boundaries in a given area. In the traditional thresholding method, a voxel is classified as either “black” or “white” for a given CT intensity threshold, as shown in Fig. 2b.

The CT histogram displays the range and frequency of the voxel intensity value, which is noted as the CT number (a dimensionless quantity) in this study. Therefore, the CT number reflects the X-ray attenuation coefficient in an image voxel. As has been frequently reported, if the image is noiseless, then all voxels in the same component exhibit the same X-ray attenuation and therefore the same CT number; this is not the case for X-ray tomographic images because of various artifacts that occur during reconstruction^23,25^. In previous studies, the detector read-out noise can be accounted in several ways such as the Poisson distribution^25^. In another study, it was reported that it is extremely difficult to specify the actual overall noise distribution, but it can be estimated that the overall observed noise is distributed normally; in addition, the intensity of voxels belonging to the pure phase (either air or solid) is distributed normally^23^. Similarly, the normal distribution of voxel intensity values for a single phase is assumed in this study.

**Figure 3:**
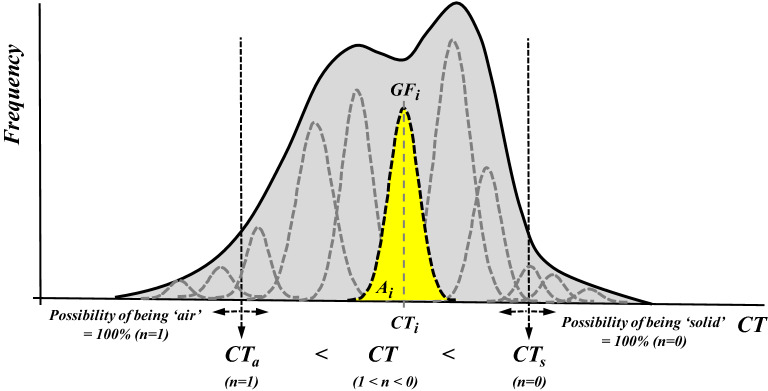
Concept of the statistical phase fraction method for a sample composed of double phases of air and solid.

In general, the SPF method proposed in this study employs a CT histogram to estimate the volume fraction of the three common phases of matter, i.e., solid, liquid and air. For the convenience of porosity analysis, both the air and liquid phases are referred to as voids and therefore, a porous material consists of voids and solids. The total porosity can be calculated as the volume fraction of the air and liquid phases. For a dry sample, only the air and solid phases exist. There are two prerequisites for the SPF method:

1. The CT histogram of a pure phase or a state of phase combination can be described by a normal distribution, i.e., a bell-shaped Gaussian function (GF), and the CT number at the peak frequency can represent the phase;
2. The CT histogram of a composite can be described by a combination of multiple GFs that have different phase fractions, as shown in Fig. 3. The summation of the area of each GF (i.e., volume of voxels corresponding to each GF) is equal to the area of the CT histogram (i.e., total volume). The portion of each phase for a given GF is determined by the relative locations (mean values) of the present GF and pure-phase GFs.

When considering a dry sample composed of two phases of air and solid, as shown in Fig. 3, the irregularly shaped CT histogram curve (in solid) can be fitted by multiple GFs with the summation of the area of each GF (*A*_i_, in yellow) being equal to the total shadow area (in gray) of the CT histogram. Suppose that the CT numbers for air and pure solid are *CT*_a_ and *CT*_s_, corresponding to void ratios of *n* = 100% and zero, respectively. Therefore, any *GF*_i_ having the mean value (*CT*_i_) between the two endmember CT numbers (*CT*_a_ and *CT*_s_) is composed of a mixed phase of air and solid, and accordingly, the volume fraction of the air and the porosity *n* (0 < *n* < 1) can be calculated as follows:

1 $$A_{a}^{i} = A_{i} \cdot\Phi_{i} = A_{i} \frac{{CT_{i} - CT_{a} }}{{CT_{s} - CT_{a} }}$$

2 $$n = \mathop \sum \limits_{1}^{N} A_{a}^{i} /\mathop \sum \limits_{1}^{N} A_{i}$$

where *N* is the total number of GFs, *A*_i_ is the area of the *i*th GF, and *Φ*_i_ is the volume fraction of air in the *i*th GF. Therefore, (1 − *Φ*_i_) is the volume fraction of the solid for the double-phase sample. Hence, ${A}_{a}^{i}$ represents the volume of air in a total of voxels having a specific *Φ*_i_.

For details about the calculation process of the SPF method for an arbitrary combination of the three phases, refer to Appendix. In this study, *CT*_a_ and *CT*_s_ are determined as two independent parameters and the Eq. (1) is formulated explicitly.

## Materials and results

### Basic properties of materials and specimens

**Figure 4:**
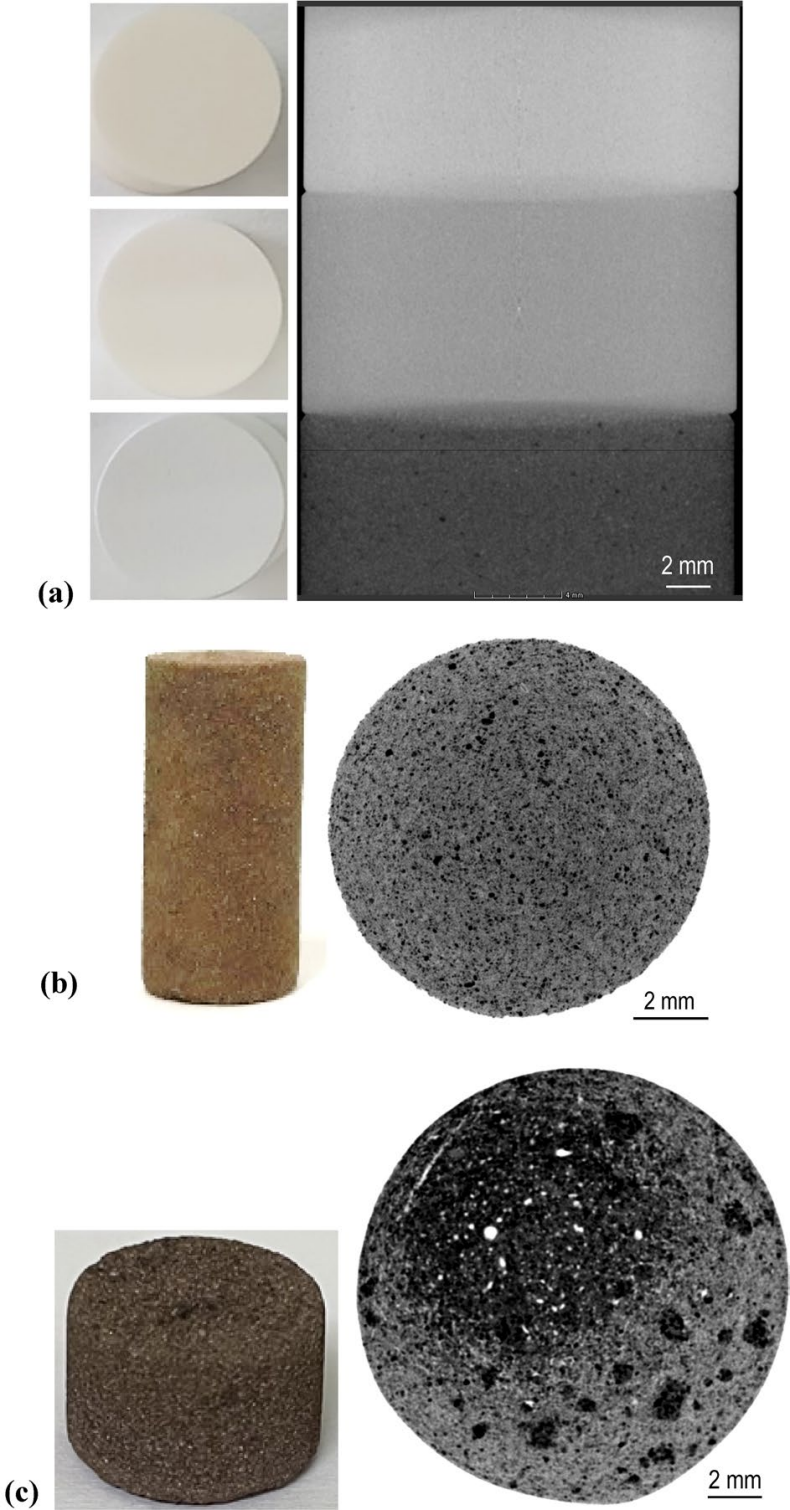
Three different types of specimens used in this study and their typical cross-sectional CT images showing microstructures. (**a**) Three alumina specimens and corresponding cross-sectional CT images with given porosities of 0% (upper), 11% (middle) and 51% (bottom), (**b**) Typical MS-KLS1 specimen and its cross-sectional CT image, and (**c**) Typical MS-FJS1 specimen (Group III) and its cross-sectional CT image, where white parts show pores and cracks.

Three types of materials and their corresponding specimens with various porosities are employed for validation of both the porosity estimation and homogeneity evaluation (Table 1). The first type of material refers to the homogenous alumina ceramic specimens in Fig. 4a with known porosities of 0%, 11% and 51%, which were calibrated by the manufacturer; they are named F136, T115 and T332, respectively. The alumina is also called aluminum oxide (Al_2_O_3_), and it has a theoretical specific mass of 3.95 g/cm^3^. Specimens F136 and T115 are made in 99.6% alumina and T332 is made in 94% alumina. The pore sizes in the alumina specimens are on the micrometer scale. The other two types of specimens are manufactured through microwave sintering (MS) using the Korean Lunar Simulant #1 (KLS-1) and Fuji Japanese Simulant #1 (FJS-1) of lunar regolith. For the KLS-1 specimen, only fine particles with grain sizes below 0.85 mm are used. The true density for the sintered KLS-1 and FJS-1 specimens is measured to be approximately 3.05 g/cm^3^ and 2.93 g/cm^3^, respectively. For details regarding the sintering process and the physical and chemical properties of the sintered specimens, we refer to previous studies^5,26,27^. The alumina specimen has a perfect cylinder shape with a diameter of 20 mm and height of 10 mm; the MS-KLS1 specimen as shown in Fig. 4b is cylindrical with a diameter of ca. 10 mm and 20 mm in height; and the last type of MS-FJS1 specimen as shown in Fig. 4c, has a near-cylindrical surface with an average diameter of 15 mm and a height in the range of 10–12 mm, depending on the sintering conditions, such as temperature, heating rate and heating duration.

**Table 1:** Three sets of specimens and their total porosities measured in the laboratory compared with the estimation results of the SPF method.

Material	Specimen No.		Bulk volume (mm^3^)	Total porosity measured in laboratory, *n* (%)	SPF estimation, *n*_SPF_ (%)	Difference, *Abs*(*n*_SPF_-*n*) (%)	Error, *Abs*(*n*_SPF_ -*n*)/*n* (%)
Alumina ceramic	F136		3141.59	0	0.13	0.13	*N/A*
	T115			11	11.06	0.06	4.09
	T332			51	50.67	0.33	0.65
Microwave sintered KLS-1	KMS1		1567.94	33.0	33.32	0.32	0.97
	KMS2		1535.02	29.12	29.08	0.04	0.13
	KMS3		1541.69	25.99	24.86	1.13	4.35
Microwave sintered FJS-1	Group I	FMS1	2564.94	33.59	33.49	0.10	0.30
		FMS2	2411.79	35.11	32.49	2.62	7.46
		FMS3	2569.47	34.61	33.54	1.07	3.09
	Group II	FMS4	2239.06	25.94	25.18	0.76	2.93
		FMS5	2307.80	27.59	26.00	1.59	5.76
		FMS6	2464.75	30.56	29.78	0.78	2.55
	Group III	FMS7	1771.04	14.46	13.11	1.35	9.34
		FMS8	1785.92	10.99	11.23	0.24	2.18
		FMS9	1967.16	14.40	14.80	0.40	2.78

Industrial X-ray cone-beam CT is used in the present study. The equipment contains three X-ray tubes operating at the maximum voltages of 120 kV, 225 kV and 320 kV, which were designed for different types of materials with a large range of densities. The tube with high voltage range of 30–225 kV is used in this study, and the minimum focal spot size is 6 μm at 5 W power and 15 μm at 25 W. The system is equipped with a flat panel detector that has an active area of 40 cm × 40 cm and a pixel matrix of 2048 × 2048. Typical cross-sectional CT images for each type of specimen are shown in Fig. 4a–c, and these images correspond to voxel sizes of 0.0319, 0.0125 and 0.027 mm for the three sets of specimens, respectively. The different voxel sizes are mainly attributed to the different source-to-object distances (SODs) used for the different specimen sizes. We confirmed from CT images that alumina specimens have a quantitatively higher level of uniformity than the other specimens. The MS specimens are mainly composed of solid (minerals), pores and a few cracks depending on the sintering conditions. Nine MS-FJS1 specimens are analyzed in this study and are divided into three groups. Each group contains three specimens created at the same sintering temperature of 1075 °C (Group I), 1100 °C (Group II) and 1125 °C (Group III). The dwell time and heating rate vary among the three specimens in the same group, and therefore porosity varies for each sintered specimen. The FMS specimens show random distribution of large pores and cracks that are visible in CT images and appear more nonuniform than the MS-KLS1 specimens, which did not show any noticeable cracks.

In addition, X-ray fluorescence (XRF) analysis results show that there are over ten chemical compositions for both the FJS-1 powder and KLS-1 powder, including mainly SiO_2_, Al_2_O_3_, Fe_2_O_3_, CaO. Additionally, the mass change in each composition before and after sintering temperature of 1075–1125 °C is insignificant^3^. This is important because chemical compositions directly influence the density of the solid matrix. Both changes in density and chemical compositions will influence CT numbers. For sintered specimens, the true density (${\uprho }_{t}$) for each sample is directly measured in the laboratory via a helium gas pycnometer. The bulk density (${\uprho }_{b}$) is estimated based on the true mass (*m*) of the sample and the bulk volume (${v}_{b}$) obtained from surface determination in CT analysis, for the microwave sintered specimens in particular. This is because the MS specimens contain a few open cracks, and Archimedes’ method is not suitable to measure the bulk volume. In addition, the bulk volume measured from CT images is also validated by a 3D laser scanner to ensure that the porosity measured in the laboratory is close to the ground truth.

3 $${\uprho }_{b} = m/v_{b} = {\uprho }_{t} \left( {1 - n} \right)$$

4 $${\uprho }_{t} = m_{s} /v_{s}$$

where *n* is the porosity and *m*_s_ and ${v}_{s}$ are the mass and volume of the solid, respectively. For a sample composed of only air and solid, *m* ≈ *m*_s_, and *m* = *m*_*l*_ + *m*_*s*_ for specimens containing a liquid phase with a mass of *m*_*l*_.

### Macropores and cracks in sintered specimens

**Figure 5:**
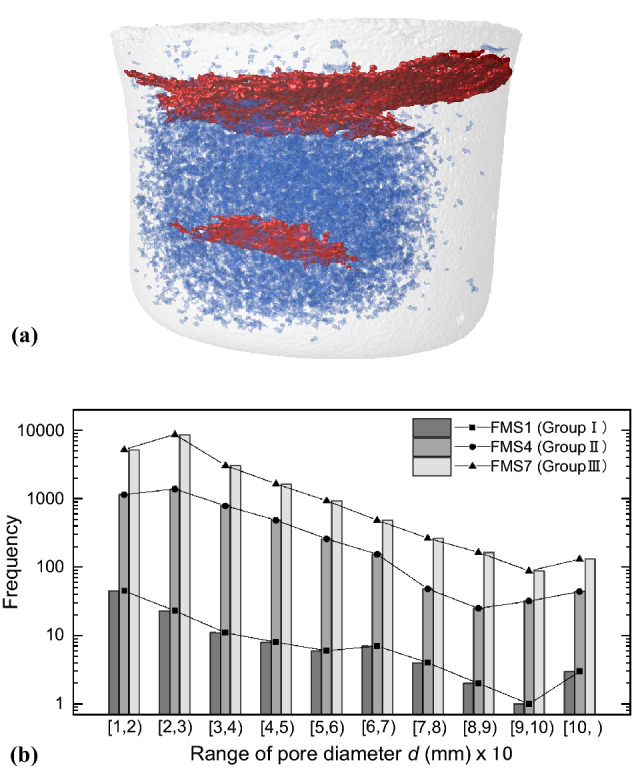
Macropores and cracks in sintered MS-FJS1 specimens. (**a**) Reconstructed structure of the FMS4 specimen (total porosity 25.94%) showing two cracks (in red) with a volume fraction of 3.25% and a few macropores (in blue) with a volume fraction of 0.79%. (**b**) Comparison of the pore size distribution for macro pore with diameter *d* ≥ 0.1 mm. Frequency means the total number of pores in the specified range of the pore size.

As confirmed from the CT images shown in Fig. 4, there are few macropores in the alumina specimens and MS-KLS1 specimens, while the sintered MS-FJS1 sample particularly the group II and the group III specimens, contains macropores and minor cracks that are visible in the CT image. We therefore conduct quantitative analysis on these macroscopic defects. Figure 5a shows a typical CT image reconstruction of the macropores and two cracks in the FMS4 specimen. Based on the segmentation, the cracks were measured to have a volume fraction of 3.25% in the total bulk volume of 2239.06 mm^3^ for the FMS4 specimen.

Figure 5b compares the pore size distribution for specimens FMS1, FMS4 and FMS7, representing Group I, Group II and Group III of the MS-FJS1 sample, respectively. The pores are detected using the porosity/inclusion analysis algorithm provided by VGStudio Max 3.2 software under the filtering condition of a probability larger than 0.8. As a result, a total of 111, 4371 and 20,717 pores with a wide range of equivalent diameters (0.1–2.5 mm) in the FMS1, FMS4 and FMS7 specimens, respectively, are detected. The number of macropores in FMS1 is negligible compared with those in the other two specimens. The Y-axis (frequency) is therefore shown on a logarithmic scale due to the large differences in the magnitudes. The total range of pore size is divided into 10 sections for the convenience of comparison of the pore size distribution, as shown in Fig. 5b, e.g., [1, 2) means the pore diameter 1 ≤ 10*d* < 2 mm. The results indicate that FMS4 (group II) and FMS7 (group III) specimens have very similar pore size distribution, showing the most macropores with diameters of 0.2 to 0.3 mm, and the number decreases across the next six sections until the diameter range of [9, 10), i.e., 0.9 ≤ *d* < 1 mm. The macropores in specimen FMS1 (group I) mostly appear in the diameter range of 0.1–0.2 mm, occupying 40.5% of the total macropores.

Compared with the bulk volume of the specimen, the macropores in the FMS1, FMS4 and FMS7 specimens have total volumes of 0.99 mm^3^, 17.76 mm^3^ and 70.03 mm^3^, corresponding to volume fractions of 0.04%, 0.79% and 3.73%, respectively. Therefore, the total volume fracture of macropores and cracks in specimen FMS4 is 4.04%, which is much smaller than the total porosity of 25.94%. There were no cracks detected in specimens FMS1 and FMS7. In general, based on the quantitative analysis of macroscopic defects, we conclude that subresolution (less than 8 voxels) pores exhibit a total volume fraction of approximately 70% to 99.9% varying in different specimens, and therefore subresolution pores dominate the pore structures of the specimens being analyzed in this study. This is the most important reason that the SPF method is applied for porosity estimation.

### Porosity estimation and comparison with laboratory measurements

The estimated porosity for each specimen by the SPF method and its laboratory measurement, and their differences are listed in Table 1. In addition, the error in the estimation is calculated for comparison. For the 15 specimens, the difference in the porosity estimation ranges from 0.02 to 2.62%. The maximum estimation error is 4.09% for alumina sample, 9.34% for the MS-FJS1 sample, and 4.35% for the MS-KLS1 sample. The average estimation error in the FMS1–FMS9 specimens is 4.04%, which is higher than the 1.82% in the KMS1–KMS3 specimens. Clearly, the preexisting macroscopic voids, i.e., pores and cracks as shown in Fig. 5a cause high inhomogeneity and significantly influence the prediction results because the CT numbers of the macro voids vary at different locations. However, as the total volume fraction of cracks ranges from 0 to 3.25%, which is considered minor, the porosity estimation by the SPF method is conducted on the whole bulk volume specimen rather than the remaining parts that exclude the cracks.

### Homogeneity evaluation

**Figure 6:**
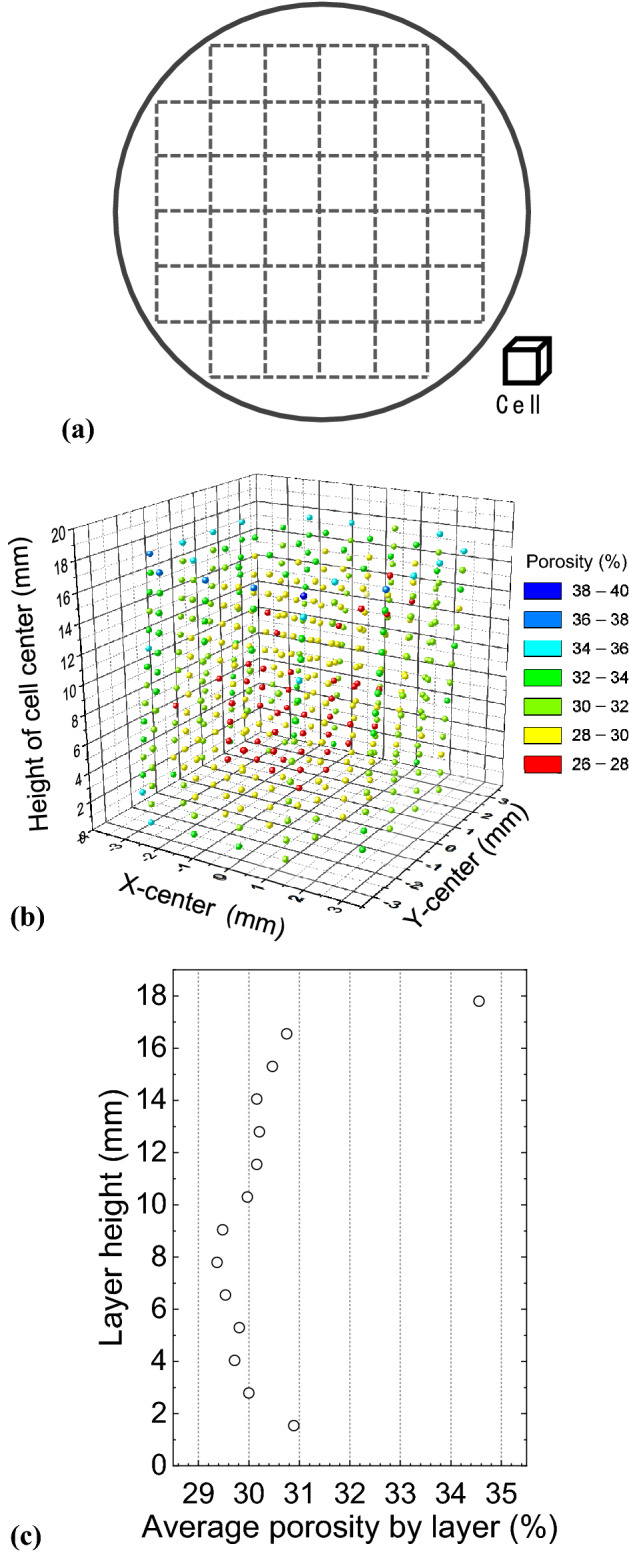
Local porosity estimation results for each cell defined in the KMS1 specimen. The X- and Y-distances are calculated by referring to the center of the specimen in the X–Y plane. Zero height is determined at the bottom plane of the specimen. (**a**) Cubic cell partition shown in the cross section, (**b**) Spatial distribution of the local porosity for the specified cell, (**c**) Average porosity in each layer, the layer thickness equals the cell size and the layer height was measured at the center of the cell referring to the zero height at the bottom of the specimen.

Many porous materials, including the sintered specimens in this study, have heterogeneous microstructures. Therefore, the total porosity is not representative of the sample. In this case, the SPF method has a large advantage in that the sample can be divided to estimate the local porosity of the sample with an arbitrarily shaped boundary, as it is easy to obtain the CT histogram of the target object once it is selected from the whole data set. Here, taking sample KMS1 as an example, we analyze the local porosity via the SPF method. The REV cell contains a total of 1E6 voxels, corresponding to a unit volume of 1.25 × 1.25 × 1.25 mm. The specimen is divided into 14 layers, and each layer contains a total of 32 cubic cells, as shown in Fig. 6a. A CT histogram for each cell is extracted, and SPF analysis is conducted on each cell to estimate its porosity.

Figure 6b shows the estimated local porosity for each cell plotted with the center coordinates, referring to the origin (0, 0) located at the center of the specimen in the X–Y plane, and zero height at the bottom plane of the specimen. In general, the upper half shows a relatively larger porosity than the lower half. The minimum and maximum local porosities are 26.25% and 38.78%, respectively. The average porosity on the 32 cells in each layer is compared in Fig. 6c, where the top layer shows a much higher porosity than the other layers, and the minimum porosity occurs at approximately the middle heights of 7–9 mm. The difference in the porosities between the top and bottom of the MS-KLS1 specimen is mainly attributed to the initial packing condition of the lunar regolith simulant. The specimen was prepared by the tapping method, and the lower part tends to be denser (as fine particles move downward) than the upper part prior to the start of sintering.

**Figure 7:**
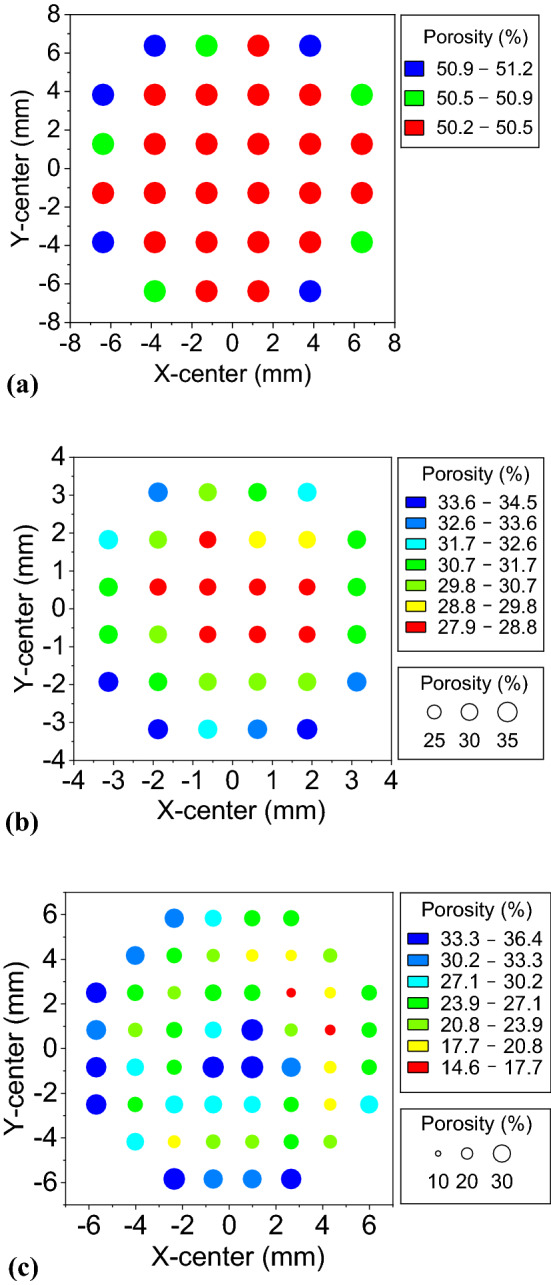
Local porosity in one specified layer in the top half. (**a**) T332 specimen (*n* = 51%), (**b**) KMS1 specimen (*n* = 33%), and (**c**) FMS4 specimen (n = 25.94%). The origin of the sample is determined as the center in the horizontal direction and the bottom in the vertical direction.

Figure 7 shows typical results of the local porosity distribution in one layer of the selected specimen that representing each set with different materials. The local porosity ranges from 50.22 to 51.13% for the specified layer in the T332 specimen in Fig. 7a, 27.88% to 34.54% in the KMS1 specimen in Fig. 7b, and 14.6% to 36.4% in the FMS4 specimen in Fig. 7c. The difference between the minimum and the maximum local porosities for the entire specimen is 2.81% (T332), 12.53% (KMS1) and 21.8% (FMS4), respectively, corresponding to approximately 6%, 38% and 84% of their total porosities. The very large difference in the local porosities of the FMS4 specimen is due to large pores and cracks, as shown in Fig. 5a. Figure 7b shows that the internal structure is denser than the external structure of the KMS1 specimen. This result is consistent with findings from SEM analysis^28^. During microwave sintering, in addition to heat convection, the sample absorbs electromagnetic energy to achieve self-heating, and the internal temperature is usually higher than the external temperature^29,30^.

The above analysis results of local porosities indicate that the two kinds of sintered specimens made in lunar regolith simulant (KLS-1 & FJS-1) are nonhomogeneous while the alumina specimens are quite homogenous. This finding is consistent with the known material information described in the Materials section. In addition, the local porosity distribution in the sintered KLS-1 and FJS-1 specimens can be well explained by the microscopic observations through CT and SEM analysis. These results demonstrate that the SPF method can be employed for homogeneity evaluation of subresolution pore-dominated porous materials.

## Discussion

Several factors possibly cause errors between the SPF estimations and the measured porosities in the laboratory. The total porosity of sintering specimens directly measured in the laboratory is only dependent on the true density and the bulk density (Eq. 3 and Eq. 4), and the errors in the laboratory measurement are recognized as insignificant in this study. Our interpretations are as follows. The true density is measured via helium gas pycnometry and is taken as the mean value of three specimens with an error of ± 0.01%^5^. The bulk density is calculated based on the bulk volume measured from CT images, and surface determination will cause an error of 1–2 pixels in the surface boundary. As a result, the error in bulk density is no more than 0.01%. In addition, the measurements of the bulk volume via CT surface determination and 3D laser scanning are compared, and the results indicate that the difference between the two measurements was insignificant. Therefore, we recognize the laboratory measurement of the total porosity for each specimen as the true value. The errors between the measurements of the two methods are thought to be mainly induced by the SPF method. In the following sections, the main factors influencing porosity estimation results are presented and discussed in the order of priority, i.e., CT numbers of the pure solid and air and artifacts in CT images.

In the SPF method, ${CT}_{a}$ and ${CT}_{s}$ (CT number for pure solid and air) are the two most important parameters that directly influence the porosity estimation result. In this study, *CT*_s_ is determined based on the approximately linear relationship between the measured porosity in the laboratory and the CT number at the peak frequency, and this value is further calibrated through standard aluminum and PMMA phantoms. The *CT*_a_ is determined to be 1840–1900 depending on the scanning case. We were not able to take a proper value for *CT*_a_ directly from CT images. The CT number for air is significantly influenced by its surrounding solid structures because the air density is three orders of magnitude lower than the solid density, e.g., the partial volume effect. However, for a given small range of densities for the same material, e.g., KMS1–KMS3 specimens, *CT*_a_ can be taken as a constant value. In addition, *CT*_a_ can be back calculated by matching the estimated porosities with the laboratory measurements. For the porosity estimation of different media, absolute values of *CT*_a_ are less important while its relative value to the pure solid should be taken seriously. We recommend further investigating the relationship between *CT*_a_ and *CT*_s_ in subresolution pore-dominated specimens with a variety of true densities, under different scanning conditions.

Second, artifacts (or noises) frequently occur during CT scanning, such as the beam hardening effect and ring artifact, which will influence the accuracy of CT numbers and image quality^31^. Beam hardening causes lower attenuation coefficients to be constructed for deeper voxels during scanning of a uniform object^32,33^. Therefore CT numbers could vary for the same phase at different locations. Because the estimation results in the SPF method are highly dependent on the selection of CT numbers, artifacts in CT images should be carefully treated. In this study, the maximum sample size is 20 mm in diameter. We investigated CT numbers along the radial direction for the zero-porosity uniform alumina specimens, and found that the CT number varies slightly along the distance; therefore, the beam hardening artifacts are not significant for the 20-mm diameter specimens analyzed in this study. In future studies, we will further validate the SPF method in sintered specimens of lunar regolith simulant with a larger size of 30–100 mm.

## Conclusions

We propose a new method, i.e., the statistical phase fraction (SPF) method, to estimate the total porosity and local porosity distribution of subresolution pores-dominated porous materials based on CT histogram analysis. In the SPF method, a voxel is treated as a mixture of two phases or three phases (i.e., air, liquid and solid). CT histogram is fitted by multiple Gaussian functions (GFs), and the mean value and the area of each GF, together with CT numbers for the pure phases, are employed to estimate the volume fraction of pores. The SPF method was validated on three different types of two-phase (i.e., air and solid) specimens that have low to high homogeneity. The estimated porosities of a total of 15 specimens are very close to the laboratory measurements via a helium gas pycnometer, showing an error range of 0–9.34% with a mean value of 3.11%. Moreover, local porosities in the defined REVs and their spatial distribution in the specimens are investigated by the SPF method. Results show that the local porosity inhomogeneity in the sintered FJS-1 specimens is more prominent than that in the sintered KLS-1 specimens.

## Methods

### Determination of CT_a_ and CT_s_

**Figure 8:**
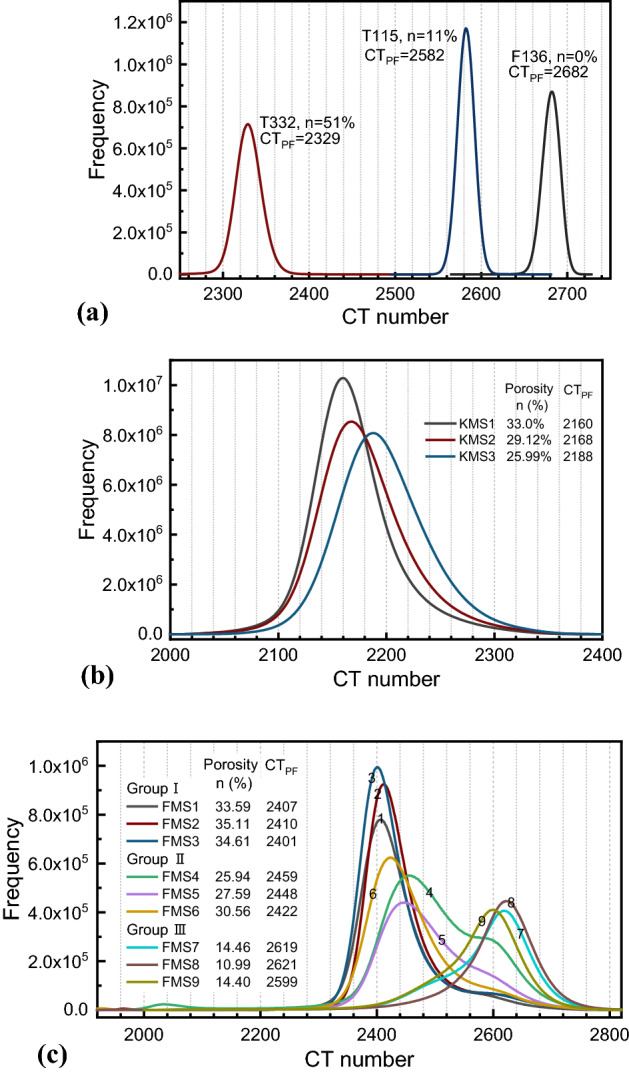
CT histogram of the three sets of specimens. (**a**) Three alumina specimens (T332, T115 and F136), (**b**) Three MS-KLS1 specimens (KMS1–KMS3), and (**c**) Nine MS-FJS1 specimens (FMS1–FMS9).

In the SPF method, ${CT}_{a}$ and ${CT}_{s}$ are the two most important parameters directly influencing the porosity estimation result. Figure 8 shows CT histograms of all three types of specimens used in this study. We found that CT histogram for each specimen shows a unimodal distribution, mainly because that the resolution is not high enough to separate two phase of pores and solid matrix. For alumina specimens with high homogeneity, CT histograms approximate a standard Gaussian distribution, and the CT number at the peak frequency (*CT*_PF_) can be recognized as the representative CT number for the matrix. In addition, there is a common trend in which *CT*_PF_ increases with decreasing porosity. For MS specimens showing significant inhomogeneity, CT histogram presents irregular patterns, showing unimodal or bimodal distributions. The presence of large cracks filled with air results in a relatively lower peak at the far left of the CT histogram, e.g., the FMS4 specimen shown in Fig. 8c. However, the main peak with the highest frequency represents the matrix.

**Figure 9:**
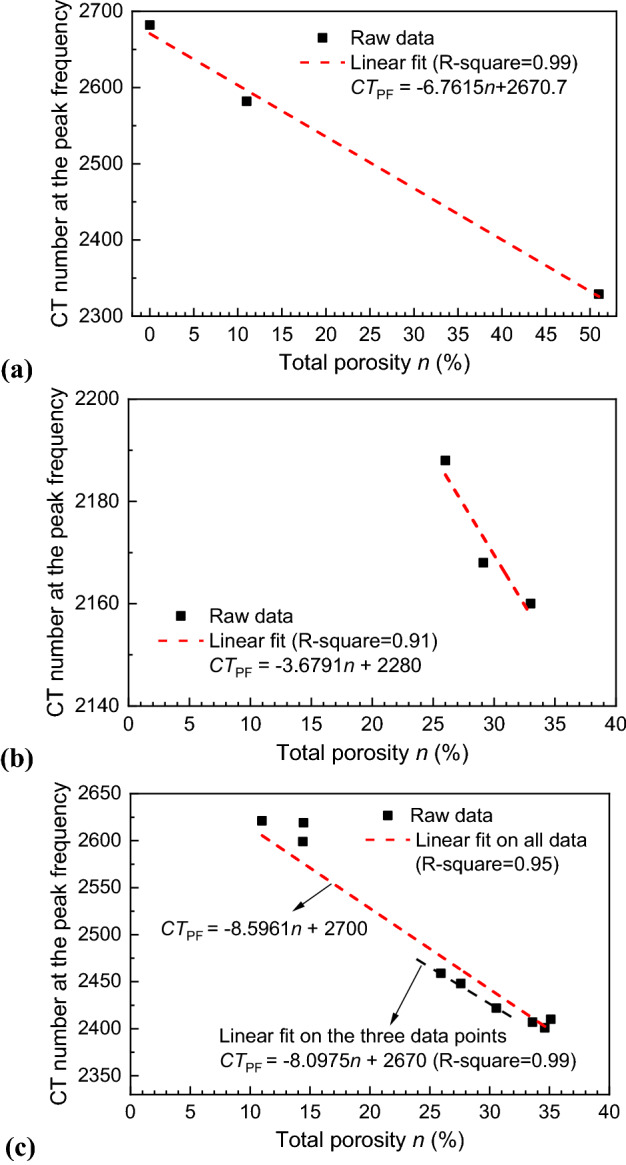
Relationship between ${CT}_{PF}$ and the total porosity. (**a**) Three alumina specimens, (**b**) three MS-KLS1 specimens, and (**c**) nine MS-FJS1 specimens.

Attenuation in CT scanning is directly related to physical density of the material. For the same material, in particular, for specimens with a uniform matrix, the CT number is approximately proportional to the bulk density and therefore the porosity^34^. Therefore, we try to obtain a linear fitting of the relationship between porosity (*n*) and *CT*_PF_ as shown in Fig. 9. The Y-axis intercept therefore corresponds to the representative CT number for the material with zero porosity (i.e., pure solid, *CT*_s_). As mentioned in the Materials section, the porosity for each specimen was either provided by the manufacturer or was measured in the laboratory beforehand. For the alumina specimens, we obtain an almost perfect linear relationship between the two parameters, while the data points of the two types of sintered specimens in Fig. 9b,c are relatively scattered. For highly nonhomogeneous materials such as the MS-FJS1 specimens employed in the present study, the number of data points and the range of porosity will influence the fitting results. The Y-axis intercept is 2670 for the linear regression with data points of the Group II only and is 2700 with data points from all nine FMS specimens.

According to Eqs. (1)–(2), *CT*_s_ and *CT*_a_ are the two important parameters required for porosity estimation. Table 2 lists the parameters used in SPF analysis, together with the main scanning conditions and image resolutions. *CT*_s_ is decided based on the fitting results of the Y-axis intercept on the *CT*_PF_ ‒ *n* relationship in Fig. 9. Theoretically, *CT*_a_ corresponds to the phase of 100% voids; however this value cannot be directly measured because the subresolution pores are impossible to separate. In addition, the CT number for air (pores) inside the specimen is greatly influenced by the solid matrix. We further investigate the CT histogram of the surrounding air in an annular region, which is 1–10 pixels away from the outside surface of the specimen (except for the top and bottom, where artifacts are more significant). We found that the CT histogram of the surrounding air is approximately normally distributed. The CT number at the peak as listed in Table 2, noted as *CT*_a_^su^, increases with increasing porosity (therefore decreasing bulk density) of the specimen for the same scanning conditions. This is the artifact caused by the reconstruction process, while the correlation between *CT*_a_^su^ and the material property (i.e., density) is not clear yet.

**Table 2:** CT numbers for the air and pure solid used in the SPF analysis. Group I: FMS1–FMS3, Group II: FMS4–FMS6, and Group III: FMS7–FMS9.

Material and Specimen No		*CT*_a_	*CT*_a_^su^	*CT*_s_	*CT*_a_*/CT*_s_	SOD (mm)	Voxel size (mm)
Alumina ceramic	F136	1860	1860	2671	0.696	80.3	0.0319
	T115	1880	1877		0.704		
	T332	1990	1930		0.745		
MS-KLS1	KMS1–KMS3	1950	1911	2280	0.855	31.5	0.0125
MS-FJS1	Group I	1900	1934	2700	0.704	68.1	0.0270
	Group II	1880	1920		0.696		
	Group III	1840	1915		0.681		

As *CT*_s_ has been determined as a fixed value for each group of specimens, *CT*_a_ was determined (i.e., back-calculation) through trial and error to achieve good agreement between the estimated porosities and the laboratory measurements, where the latter is recognized as the ground truth. Table 2 lists the *CT*_s_ and *CT*_a_ used in the SPF analysis for different specimens that were scanned under different conditions. The SOD and voxel size are also listed for information. In addition, the CT numbers of the surrounding air at the outer surface of the specimen are investigated. It is found that in most cases, *CT*_a_ determined for SPF estimation is different from *CT*_a_^su^, except for the two cases of alumina specimens with 0 and 11% porosity, where *CT*_a_ is almost equal to *CT*_a_^su^. The ratio of *CT*_a_ and *CT*_s_ is approximately 0.681–0.855 in this study. In addition, for each set of alumina and MS-FJS1 specimens, we found that there is a good linear relationship between the *CT*_a_*/CT*_s_ ratio selected in this study and the porosity of specimen. This means, when assuming the same CT number for the pure solid for the same material, the higher porosity of the specimen will cause smaller CT number for air (therefore, a smaller *CT*_a_*/CT*_s_). However, we have not been able to provide a sound basis to determine *CT*_a_*/CT*_s_ in this study.

### Calibration of ${\mathbf{C}\mathbf{T}}_{\mathbf{s}}$ using standard phantoms

A previous study suggested a method to employ reference materials, e.g., aluminum phantoms, ethanol and other pure substances with a given molecular formula, to establish a relationship between CT numbers and X-ray attenuation coefficients under certain scanning conditions^20^. The CT number or the gray intensity value in CT images has a linear relationship with the attenuation coefficient. The linear attenuation coefficient ($\mu$, length^−1^) is energy-dependent and can be written as:

5 $$\mu (\varepsilon ) = a(\varepsilon ) \times CT + b(\varepsilon )$$

where *a*($\varepsilon$) and *b*($\varepsilon$) are energy-dependent constants and *CT* is the CT number for each voxel. The mass attenuation coefficient (${\mu }_{s}$, length^2^/mass) equals the ratio of the attenuation coefficient ($\mu$, length^−1^) and the mass density ($\rho$, mass length^−3^). $\mu /\rho$ is a function of photon energy, and its measurement for each chemical element and compound can be referred to in the standard provided by NIST^21^. For a mixture or compound assumed to be homogenous, ${\mu }_{s}$ can be estimated as

6 $$\mu_{s} = \mu /\rho = \mathop \sum \limits_{i} w_{i} \left( {\mu /\rho } \right)_{i}$$

where ${w}_{i}$ and ${(\mu /\rho )}_{i}$ are the weight fraction and mass attenuation coefficient of the *i*th constituent element, respectively.

**Figure 10:**
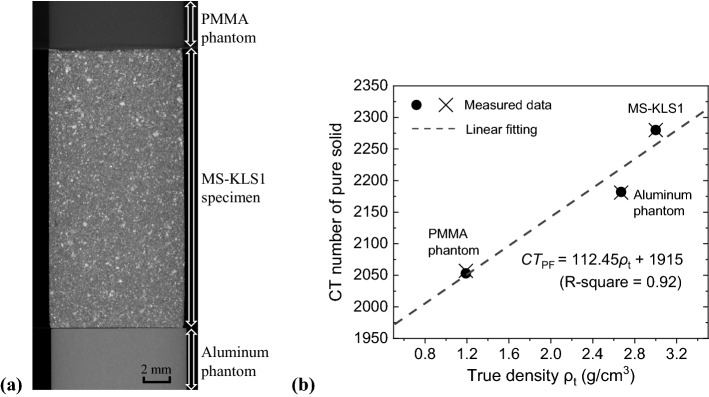
CT scanning of the specimens and specified phantoms. (**a**) Typical cross-sectional CT image, and (**b**) Relationship between the CT number for the solid and the true density.

The photon energy-dependent constants (i.e., *a* and *b*) in Eq. (5) can be estimated by scanning any two or more theoretically pure materials, e.g., air and aluminum, PMMA and water, or pure ethanol and aluminum, because the attenuation coefficient at given photon energy for the pure material is available in the NIST database or its theoretical value can be calculated once the chemical formula is known. Phantoms made in various materials can be used for CT number calibration for a specific CT imaging system^35^. When the two constants are determined at a given scanning condition, CT numbers for a specific phase or medium (e.g., air and pure solid) can be obtained. This method has been reported in previous studies^20,22^. In this study, we employ PMMA and aluminum phantoms with true densities of 1.19 g/cm^3^ and 2.67 g/cm^3^, respectively, to calibrate CT numbers for given scanning conditions (SOD 31.5 mm, voltage 120 kVp, current 200 μA), as shown in Fig. 10. The two phantoms have the same diameter of 10 mm as the MS-KLS1 specimen, and the three specimens are stacked in the order of low to high densities to reduce the cone beam artifacts appeared between the high and low density regions. The CT histogram for phantoms composed of pure solids shows a nearly perfect normal distribution, and the CT number for pure solids is easily obtained at ${CT}_{PF}$. The true density of the MS-KLS1 specimen is ~ 3.0 g/cm^3^, and ${CT}_{s}$=2280 is determined according to Fig. 10b. A good linear fitting between the true density and CT number for the three types of pure solids is obtained, as shown in Fig. 10b. This result has shown that the ${CT}_{s}$= 2280 used in the SPF analysis on the three MS-KLS1 specimens is reasonable, and the calibration method using phantoms is effective. However, this method has limitations, as CT numbers for phantoms are also influenced by scanning conditions such as the SOD and phantom size. To obtain the most reliable results, the phantoms and the analyzed specimen should have similar sizes in the direction parallel to the X-ray beam, and therefore they should be scanned at the same SOD.

### Gaussian function fitting

**Figure 11:**
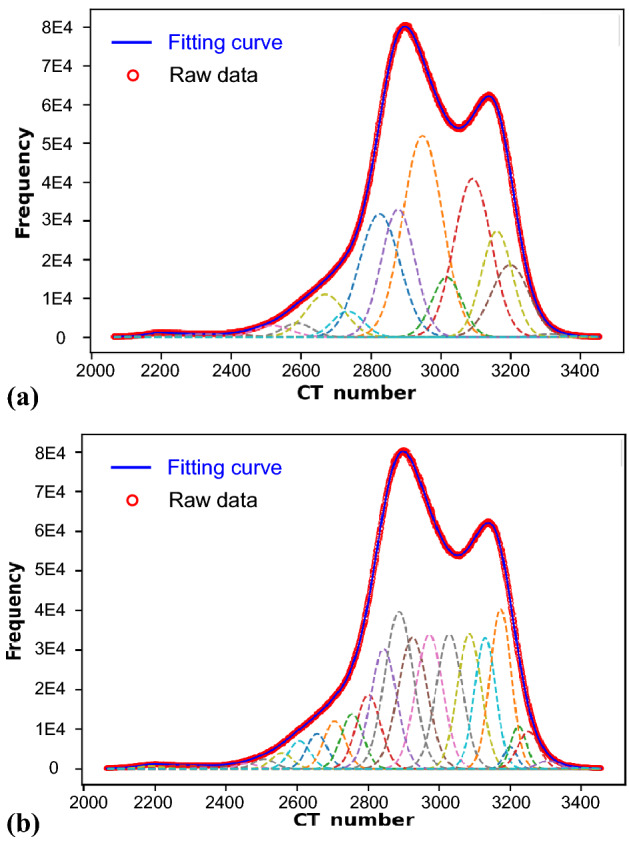
Comparison of the fitting results using (**a**) 20 GFs with main GFs having a mean value close to the peak in the raw CT histogram data and (**b**) 30 randomly distributed GFs.

The total number of GFs and the allowed variance between the fitting results and the raw data of the frequency of CT number are determined before the start of fitting on the CT histogram. In general, a relatively large number of GFs are needed to achieve a good fitting result if the CT histogram is deviated far from the normal distribution. The number (e.g., 10 or 20 GFs) can be easily determined after several attempts. Provided that the fitted curve fits the raw data well, the total area of the GFs is very close to its ground truth; as a result, the effect of the total number of GFs on the estimated porosity is insignificant. Another factor is the algorithm used for the fitting. For example, we can employ a main GF with a mean value that is close to the value at the peak of the original data, and the other GFs will be distributed at both sides of the main GF to achieve the best fitting results; otherwise, all the GFs can be randomly distributed. Figure 11 shows an example of fitting results using two different fitting methods and different numbers of GFs. The root mean square errors (RMSEs) are 169.57 for the fitting with 20 GFs in Fig. 11a and 151.76 for the fitting with 30 GFs in Fig. 11b. The porosity estimated by the SPF method using the fitting results in Fig. 11a,b is 22.14% and 22.3%, respectively. Therefore, we confirmed that either the number of GFs or the fitting algorithm has an insignificant influence on the porosity estimation, provided that the fitting curve of the CT histogram matches well with the raw data. After the GF fitting has been implemented, the mean value (*CT*_i_) and the area of each GF (*A*_i_) in Eq. (1) are obtained.

## Data availability

All data used during the study are included in this article or are available from the corresponding authors upon reasonable request.

## Appendix: Assumption and calculation process of the SPF method

The SPF method is based on assumptions that each pure or single phase corresponds to a normal distribution of the CT histogram, and the CT number at the peak frequency can represent the phase. The calculation process in the SPF method to estimate the porosity and to conduct homogeneity evaluation is divided into four steps. Currently, this method is mainly applied in porous materials that contain a maximum of three pure phases, e.g., air, liquid and solid. The liquid can be either water or contrast medium (e.g., ethanol, brine, KI solution, oil) that are frequently used in medicine and engineering.

### Step 1: Fitting of the CT histogram by multiple Gaussian functions

A1 $$CT histogram = \mathop \sum \limits_{j = 1}^{N} f\left( {_{j} ,w_{j} } \right)$$

where *f*(${\mu }_{j},{w}_{j}$) is the *j*th Gaussian function, with its under area noted as ${GF}_{j}$, *μ* & *w* are the mean & variance of the Gaussian function, respectively; and *N* is the total number of GFs.

### Step 2: Calculate the phase ratio (PR)

Phase ratios in a specific GF are determined according to the relative positions between the GF and the positions of GFs for pure phases. For a given *j*th GF,

A2 $$PR_{i}^{j} = \frac{{\left| {\mu_{i} - \mu_{j} } \right|{ }}}{{\mathop \sum \nolimits_{i = 1}^{NP} \left| {\mu_{i} - \mu_{j} } \right|}}$$

where *i* is the current pure phase, i.e., either the *P*1 or *P*2 or *P*3 phase, and ${\mu }_{i}$ is the mean value of the GF that represents the *i*th pure phase, i.e., the CT number for the pure phase. ${\mu }_{j}$ is the mean value of the *j*th GF. *NP* is the number of pure phases; *NP* = 2 for the two-phase, and *NP* = 3 for the three-phase samples. |*μ*_i_ ‒ *μ*_j_| refers to the distance between the mean of the GF representing the current pure phase (*i*) and the *j*th GF.

The CT number for pure solids (*CT*_*s*_) can be determined using the two methods introduced in this study.

### Step 3: Calculate the volume fraction of the pure phases

Total volume fraction for the current pure phase (*i*)

A3 $$VF_{i} = \frac{{\mathop \sum \nolimits_{j = 1}^{N} PR_{i}^{j} \times GF_{j} }}{{\mathop \sum \nolimits_{i = 1}^{NP} \mathop \sum \nolimits_{j = 1}^{N} PR_{i}^{j} \times GF_{j} }}$$

where *VF*_i_ is the total volume fraction of the *i*th pure phase and ${GF}_{j}$ is the area of the *j*th GF.

For a two-phase (*NP* = 2) sample composed of air and solid, the volume fraction of air (*VF*_a_) is equal to the total porosity of the sample. For a three-phase (*NP* = 3) sample composed of air, fluid and solid, the total porosity (*n*) of the sample can be estimated as the summation of the volume fraction of fluid (*VF*_f_) and air:

A4 $$n = VF_{{\text{f}}} + VF_{{\text{a}}}$$

### Step 4: Local porosity estimation for evaluation of specimen homogeneity

First, a reasonable unit cell size (e.g., REV) is determined, and meshing of the specimen is performed to obtain an individual CT histogram corresponding to each cell. Second, SPF analysis for each individual cell is conducted to obtain the local porosity in the cell. The spatial distribution of local porosities and subsequent data analysis (e.g., difference in local porosities, average porosity by layer) are used for evaluation of specimen homogeneity.
